# RETRACTED: Srivastava et al. Match-Level Fusion of Finger-Knuckle Print and Iris for Human Identity Validation Using Neuro-Fuzzy Classifier. *Sensors* 2022, *22*, 3620

**DOI:** 10.3390/s26113386

**Published:** 2026-05-27

**Authors:** Rohit Srivastava, Ved Prakash Bhardwaj, Mohamed Tahar Ben Othman, Mukesh Pushkarna, Anushree, Arushi Mangla, Mohit Bajaj, Ateeq Ur Rehman, Muhammad Shafiq, Habib Hamam

**Affiliations:** 1 School of Computer Science, University of Petroleum and Energy Studies, Dehradun 248007, India; r.srivastava@ddn.upes.ac.in (R.S.); vpbharadwaj@ddn.upes.ac.in (V.P.B.); 2 Department of Computer Science, College of Computer, Qassim University, Buraydah 51452, Saudi Arabia; 3 Department of Electrical Engineering, GLA University, Mathura 281406, India; mukesh.pushkarna@gla.ac.in; 4 Department of Computer Engineering and Applications, GLA University, Mathura 281406, India; anushree.gla@gla.ac.in; 5 Mamaearth Pvt Ltd., Gurgaon 122001, India; arushi.m@mamaearth.in; 6 Department of Electrical and Electronics Engineering, National Institute of Technology, Delhi 110040, India; mohitbajaj@nitdelhi.ac.in; 7 College of Internet of Things Engineering, Hohai University, Changzhou 213022, China; ateeq@hhu.edu.cn; 8 Faculty of Engineering, Université de Moncton, Moncton, NB E1A3E9, Canada; habib.hamam@umoncton.ca; 9 Department of Information and Communication Engineering, Yeungnam University, Gyeongsan 38541, Republic of Korea; 10 International Institute of Technology and Management, Libreville BP1989, Gabon; 11 Spectrum of Knowledge Production and Skills Development, Sfax 3027, Tunisia; 12 Department of Electrical and Electronic Engineering Science, School of Electrical Engineering, University of Johannesburg, Johannesburg 2006, South Africa

The Journal retracts the article “Match-Level Fusion of Finger-Knuckle Print and Iris for Human Identity Validation Using Neuro-Fuzzy Classifier” [1] cited above.

Following publication, concerns were brought to the attention of the Editorial Office regarding the presence of unusual phrases in this article [1].

In accordance with standard journal procedures, the Editorial Office and Editorial Board conducted an investigation that identified further issues related to the authorship contributions and misattribution of multiple references. Although the authors cooperated with the investigation, the explanations and supporting materials provided were not deemed sufficient by the Editorial Board to resolve the identified concerns. Consequently, the Editorial Board has lost confidence in the reliability of the findings and decided to retract this publication [1], as per MDPI’s retraction policy.

This retraction was approved by the Editor-in-Chief of the journal *Sensors*.

The authors have been informed of this retraction. Rohit Srivastava, Ved Prakash Bhardwaj, Mohamed Tahar Ben Othman, Mukesh Pushkarna, and Mohit Bajaj have indicated their disagreement with this decision. The other authors did not provide a comment on this decision.
